# Metallurgical Parameters Controlling the Eutectic Silicon Charateristics in Be-Treated Al-Si-Mg Alloys

**DOI:** 10.3390/ma9020078

**Published:** 2016-01-27

**Authors:** Mohamed F. Ibrahim, Emad M. Elgallad, Salvador Valtierra, Herbert W. Doty, Fawzy H. Samuel

**Affiliations:** 1 Département des Sciences appliquées, Université du Québec à Chicoutimi, Chicoutimi, QC G7H 2B1, Canada; mohmed.ibrahim@uqac.ca (M.F.I.); emad.elgallad@uqac.ca (E.M.E.); 2 Corporativo Nemak, S.A. de C.V., P.O. Box 100, Garza Garcia, Nuevo León 66221, Mexico; Salvador.valtierra@nemak.com; 3 Materials Engineering, General Motors, 823 Joslyn Avenue, Pontiac, MI 48340, USA; herb.doty@gm.com

**Keywords:** aluminum alloys, additives, eutectic structure, fractography, tensile properties, image analyses

## Abstract

The present work was carried out on Al-7%Si-0.4%Mg-X alloy (where X = Mg, Fe, Sr or Be), where the effect of solidification rate on the eutectic silicon characteristics was investigated. Two solidification rates corresponding to dendrite arm spacings (DAS) of 24 and 65 μm were employed. Samples with 24 μm DAS were solution heat-treated at 540 °C for 5 and 12 h prior to quenching in warm water at 65 °C. Eutectic Si particle charateristics were measured using an image analyzer. The results show that the addition of 0.05% Be leads to partial modification of the Si particles. Full modification was only obtained when Sr was added in an amount of 150–200 ppm, depending on the applied solidification rate. Increasing the amount of Mg to 0.8% in Sr-modified alloys leads to a reduction in the effectiveness of Sr as the main modifier. Similar observations were made when the Fe content was increased in Be-treated alloys due to the Be-Fe interaction. Over-modification results in the precipitation of hard Sr-rich particles, mainly Al_4_SrSi_2_, whereas overheating causes incipient melting of the Al-Cu eutectic and hence the surrounding matrix. Both factors lead to a deterioration in the alloy mechanical properties. Furthermore, the presence of long, acicular Si particles accelerates the occurrence of fracture and, as a result, yields poor ductility. In low iron (less than 0.1 wt%) Al-Si-Mg alloys, the mechanical properties in the as cast, as well as heat treated conditions, are mainly controlled by the eutectic Si charatersitics. Increasing the iron content and, hence, the volume fraction of Fe-based intermetallics leads to a complex fracture mode.

## 1. Introduction

The eutectic silicon particle characteristics in Al-Si alloys play an important role in determining the mechanical properties of the alloys. Under normal conditions, the eutectic Si particles display an acicular or lamellar morphology. Strontium is commonly used in Al-Si casting alloys to modify the morphology of the eutectic Si from a coarse, flake-like form to a fine fibrous one, so as to improve the mechanical properties, in particular, the ductility [1,2,3].

With the addition of Sr, the eutectic temperature of the Al-Si eutectic reaction is depressed. Consequently, this depression is often used to estimate the degree of modification which has taken place in the Al-Si alloy. Other alloying elements, such as magnesium and beryllium, together with varying amounts of iron, manganese and zinc as impurity elements, form intermetallic particles during solidification [4,5].

When combined with Sr, Mg negates the effect of Sr modification to such an extent that a much higher level of Sr is required to achieve full modification of the eutectic Si structure. In the Sr-modified Mg-free alloy, the Al-Si eutectic is better modified than the one in the Mg-containing alloy modified using the same amount of Sr. The volume fraction of the Fe-rich intermetallics in 357 alloys is larger than it is in 356 alloys containing the same level of Mg as a result of the formation of larger amounts of the *π*-AlFeMgSiFe-intermetallic phase [6,7].

Heat-treatable aluminum alloys are those whose mechanical properties may be improved by means of a specific heat treatment. The T6 heat treatment process involves three stages, namely, solution heat treatment, quenching and aging. The purpose of the solution heat treatment is to place the maximum amount of hardening solutes, such as Mg, into solid solution in the aluminum matrix [8,9,10].

The recommended solution temperature for 356 and 357 alloys is 540 ± 5 °C, as this permits maximum concentrations of Mg and Si in solid solution. Quenching is then carried out, and is usually conducted in water, making it possible to freeze the structure for a brief period of time. The purpose of this process is to preserve the solid solution formed at the solution heat-treating temperature by means of rapid solidification to some lower temperature usually close to room temperature [11,12,13].

Samuel *et al*. [14] studied the effect of melt cleanliness on the properties of an Al-10 wt%Si-10 vol%SiC composite. Their finding show that inclusions and associate microvoids act as the crack initiation sites during composite fracture. Simple filtration using 10 ppi ceramic foam filters under gravity serves adequality in removing these inclusions and producing the desired mechanical properties. It has been proposed that, effectively, there are two different Al-Si eutectic reactions. During solidification from the liquid state, because oxide films are such favored substrates for most intermetallics, the silicon first forms and grows on bifilms, explaining the large flake-like morphology of this silicon, and its apparent (not real) brittleness because of the bifilm crack down its center, and its consequential rather poor mechanical properties. Remaining silicon in solution, if any, then nucleates on some other (currently unknown) substrate at significantly lower temperatures, and grows as a classical coupled eutectic—the silicon taking the form of a continuous fine flake-like phase or continuous coral morphology [15,16].

Campbell [17] proposed that tangled thin oxides created during the filling of a mould become flattened or acquire a spherical shape because of several processes: the penetration of dissolved hydrogen from the liquid aluminium into the bifilm during solidification, the pushing of oxides by dendrites during solidification, the growth of heterogeneously nucleated intermetallic faces up on the wetted oxide side, and shrinkage that pulls oxides. The addition of Sr seems to chemically deactivate the bifilms as substrates, causing all the silicon to precipitate at the lower temperature as a classical eutectic. “Modification”, therefore, is a mechanism which converts Si growth on randomly scattered oxide bifilms of random sizes to classical solidification of a eutectic whose fine and regular spacing is controlled by diffusion and interfacial energy [17]. In Be-containing alloys, the silicon particles are smaller, their aspect ratio is closer to 1, and the small nodular iron-bearing compounds can improve the tensile properties. The effect on decreasing mechanical properties damage is more apparent in the higher Fe level than in the lower Fe content when Be is added [18].

The present work revivews the factors controlling the eutectic Si chateristics in relation to alloy mechanical propertes and fracture mechanism in hypo-eutectic Al-Si-Mg alloys.

## 2. Experimental Procedure

The alloys used in this work were prepared using an electrical resistance furnace having a large 40 kg crucible capacity. About 35 kg of each alloy was prepared. The melting temperature was maintained at 750 ± 5 °C. At this temperature, measured additions of Mg, Fe, Be, Sr and Ti were made to the melt by means of a perforated graphite bell. Prior to casting, the molten metal was degassed using pure, dry argon. In present case the degassing was performed using rotary graphite impeller at speed of 150 rpm for 15 min, to remove the hydrogen and inclusions.

In addition to the required number of tensile test bar castings prepared from each melt, one sampling for thermal analysis purposes and two samplings for chemical analysis—one before the start of casting and one at the end of casting—were also taken from each melt. The chemical analysis was carried out using arc spark spectroscopy at the General Motors facilities in Milford, NH, USA. The actual chemical composition of each of the alloys prepared is shown in Table 1.

**Table 1:** Nominal chemical composition (wt%) of the 356 and 357 alloys studied.

Alloy Code	Element Concentration (wt%)						
	Si	Fe	Mg	Ti	Sr	Be	Al
A1	7.146	0.09	0.40	0.168	0.00	0.00	Balance
A1B	7.146	0.09	0.40	0.168	0.00	0.05	Balance
A1S	7.146	0.09	0.40	0.168	0.02	0.00	Balance
A1BS	7.146	0.09	0.40	0.168	0.02	0.05	Balance
C3	7.146	0.60	0.80	0.168	0.00	0.00	Balance
C3B	7.146	0.60	0.80	0.168	0.00	0.05	Balance
C3S	7.146	0.60	0.80	0.168	0.02	0.00	Balance
C3BS	7.146	0.60	0.80	0.168	0.02	0.05	Balance

A and C correspond to the Fe levels 0.09 and 0.6, respectively; while codes 1,2 and 3 correspond to the Mg levels 0.4, 0.6 and 0.8, respectively; B and S represent Be and Sr, respectively.

In order to obtain the solidification curves, as well as to identify the main reactions and corresponding temperatures occurring during the solidification of Al-Si-Mg alloys, thermal analysis was carried out for all of the compositions prepared. The molten metal for each composition was poured into a cylindrical graphite mold of 80 mm height and 60 mm diameter which had been preheated to 600 °C so as to create a slow solidification rate resembling equilibrium conditions, to facilitate identifying the phases formed. A high sensitivity type-K (chromel-alumel) thermocouple, which had been insulated using a double-walled ceramic tube, was attached to the centre of the graphite mold. The temperature-time data was collected using a high-speed data acquisition system linked to a computer. From this data, the solidification curves and the corresponding first derivative curves for the different alloys were plotted to identify the main reactions occurring during solidification with the corresponding temperatures. Cylindrical specimens, 15 mm deep, were sectioned off from the centre of each graphite mold casting close to the thermocouple tip. Samples measuring 25 mm × 25 mm in cross-section were machined from these specimens, mounted in bakelite and polished following standard procedures, for qualitative and quantitative microstructural analysis. Table 2 presents the definitions of the Si particle parameters.

**Table 2:** Definition of the Si particles used in the present work.

Feature	Definition
Average Length (μm)	longest measurement of each particle
Average Area (μm^2^)	area of each particle
Roundness (percentage)	degree or percentage to which a particle is round or spherical.
Aspect Ratio	longest length measurement of a particle divided by the smallest length of the particle

High solidification rates were achieved by casting tensile test bars. The degassed molten metal was carefully poured into an ASTM B-108 [19] permanent mold preheated to 450 °C, to obtain castings for tensile testing. At the bottom of the pouring cup a ceramic foam filter (10 ppi) was placed to prevent inclusions and oxide films from entering the mold. This mold and casting set-up used to prepare castings provided two test bars, each with a gauge length of 50 mm and a cross-sectional diameter of 12.8 mm

Test bars of 356 and 357 alloys prepared for each alloy composition were divided into three sets; one set was kept in the as-cast condition; one set was solution heat-treated at 540 °C for 5 h, then quenched in warm water at 65 °C and maintained in the solution heat-treated condition; one set was solution heat-treated at 540 °C for 12 h, then quenched in warm water at 65 °C and maintained in the solution heat-treated condition. Tensile testing was carried out for the as-cast and the heat-treated test bars at room temperature using an MTS Servohydraulic mechanical testing machine (MTS, Eden Prairie, MN, USA) working at a strain rate of 1.0 × 10^−4^ s^−1^. The elongation of the test specimens was measured using an MTS strain gauge extensometer (MTS, Eden Prairie, MN, USA) attached to the specimen during the tension test. A data acquisition system was attached to the MTS machine to provide the results of the tensile test. For each sample tested a stress-strain curve was obtained to illustrate the mechanical behavior of each specimen under the loads applied. The tensile test results obtained from testing a specific specimen present the data pertaining to elongation to fracture, yield strength at 0.2% offset strain and ultimate tensile strength.

Samples for microstructural analysis were prepared from the castings obtained from the thermal analysis experiments and also from tensile-tested bars ~10 mm below the fracture surface. In the latter case, the samples were examined in the as-cast and solution heat-treated (540 °C/5 h and 540 °C/12 h) conditions. The microstructures of the polished sample surfaces were examined using an Olympus optical microscope (Olympus Canada Inc., Richmond Hill, ON, Canada) linked to a Clemex image analysis system (Clemex Technologies Inc., Longueuil, QC, Canada). Phase identification was carried out using an Electron Probe Micro-Analyzer (EPMA) in conjunction with energy dispersive X-ray analysis (EDX) and wavelength dispersive spectroscopic analysis (WDS) where required, integrating a combined JEOL JXA-8900l WDS/EDX microanalyzer (JEOL Canada Inc., Saint-Hubert, QC, Canada) operating at 20 kV and 30 nA, where the size of the spot examined was ~1 μm. Fracture surfaces were examined using Hitachi SU-8000 field emission-scanning transmission electron microscope (FE-STEM, Hitachi High-Technologies Corporation, Tokyo, Japan).

## 3. Results and Discussion

### 3.1. Effect of Solidification Rate

#### 3.1.1. Low Solidification Rate (DAS ~ 65 μm)

Samples for microstructural analysis were sectioned from the castings obtained from the thermal analysis experiments carried out for each alloy melt composition. Cylindrical specimens, 15 mm thick were sectioned off from the center of each graphite mold casting close to the thermocouple tip for all of the compositions obtained. From these, samples (25 mm × 25 mm), were machined, mounted and polished for preparation of metallographic samples for qualitative and quantitative microstructural analysis.

The Si particle characteristics for the various alloy compositions investigated are summarized in Table 3. It can be seen that after Sr-modification, the Si particle area decreased from 76.70 to 3.18 μm^2^, the Si particle length decreased from 21.60 to 3.05 μm, the aspect ratio decreased from 3.52 to 2.11, while the roundness increased from 28.4% to 46.0% in the alloy A1S sample. In the case of Be addition (alloy A1B), the Si particle area decreased to 49.60 μm^2^, the Si particle length to 15.20 μm, the aspect ratio to 2.85, while the roundness ratio increased to 33.8%. The presence of both Sr and Be in alloy A1BS resulted in decreasing the Si particle area to 2.75 μm^2^, the Si particle length to 3.00 μm, the aspect ratio to 2.18 and increasing the roundness to 43.5%. After increasing Mg and Fe contents (alloy C3), the Si particle area decreased to 20.70 μm^2^, the Si particle length to 9.63 μm, the aspect ratio to 2.86, while the roundness increased to 36.2%. The combined effect of the four elements (alloy C3BS) resulted in decreasing the Si particle area to 3.38 μm^2^, the Si particle length to 3.27 μm, the aspect ratio to 2.15 and increasing the roundness to 43.9%.

**Table 3:** Silicon particle measurements for the as-cast 357 alloys using a graphite mold with a DAS of 65 μm.

Alloy (Condition)	Area (μm^2^)		Length (μm)		Roundness (%)		Aspect Ratio	
	Av.	SD	Av.	SD	Av.	SD	Av.	SD
A1 (non-modified)	76.70	71.50	23.60	16.30	28.4	21.8	3.52	4.54
C3 (non-modified)	20.70	22.70	11.63	7.98	36.2	21.4	2.86	5.84
A1B (Be-modified)	49.60	52.40	15.20	12.00	33.8	21.8	2.85	3.45
C3B (Be-modified)	13.60	14.30	6.75	5.08	43.7	22.2	2.27	1.15
A1S (Sr-modified)	3.18	3.21	3.05	2.16	46.0	19.2	2.11	2.14
C3S (Sr-modified)	4.48	4.78	3.82	3.07	43.4	21.5	2.46	3.87
A1BS (Be + Sr-modified)	2.75	2.94	3.00	2.38	43.5	19.5	2.18	2.25
C3BS (Be + Sr-modified)	3.38	3.97	3.27	2.69	43.9	19.8	2.15	1.32

Codes A and C correspond to the Fe levels 0.09 and 0.6, respectively; while codes 1 and 3 correspond to the Mg levels 0.4 and 0.8, respectively; as well as both codes B and S represent Be additions and Sr modification, respectively; Av. = average; and SD = standared deviation.

**Figure 1:**
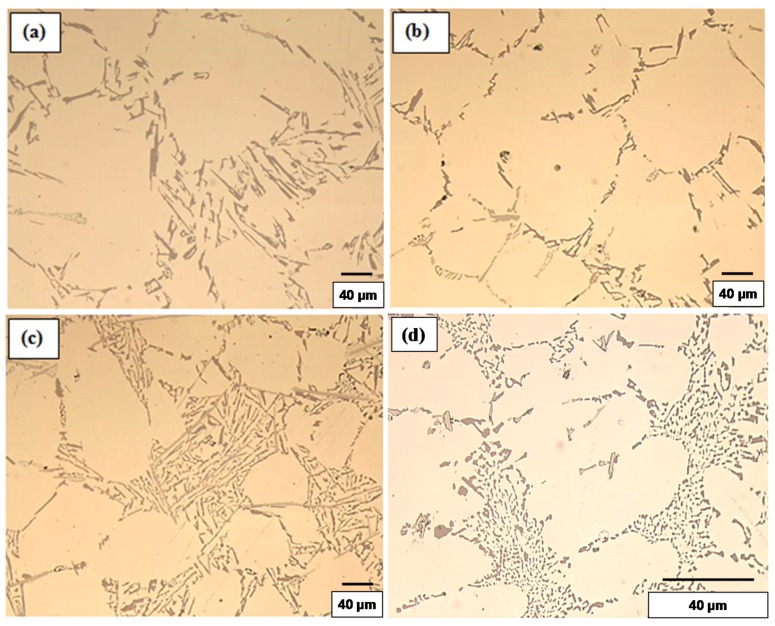
Silicon particle morphology and distribution in: (**a**) A1; (**b**) A1B; (**c**) A1S; and (**d**) A1BS alloys, DAS ~ 25 μm.

**Figure 2:**
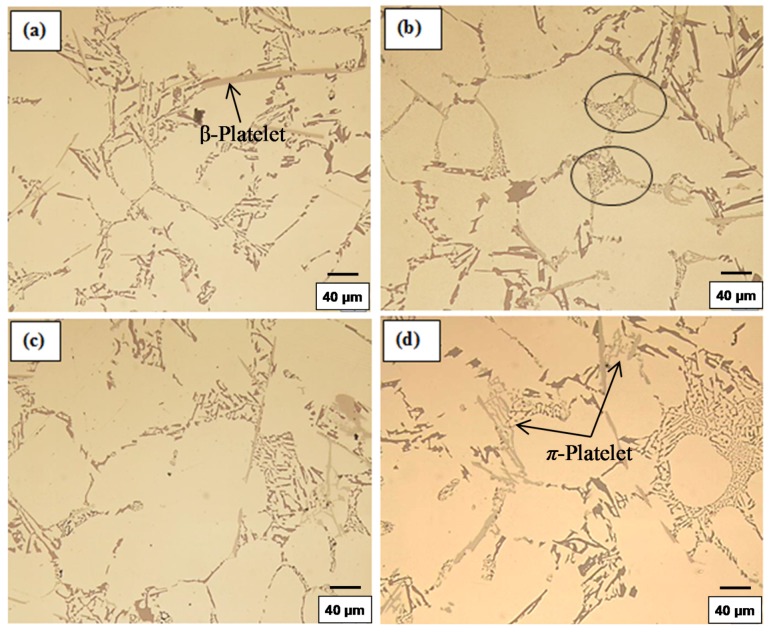
Silicon particle morphology and distribution in: (**a**) C3; (**b**) C3B; (**c**) C3S; and (**d**) C3BS alloys, DAS ~ 25 μm; fine eutectic Si particles are circled in (**b**).

**Figure 3:**
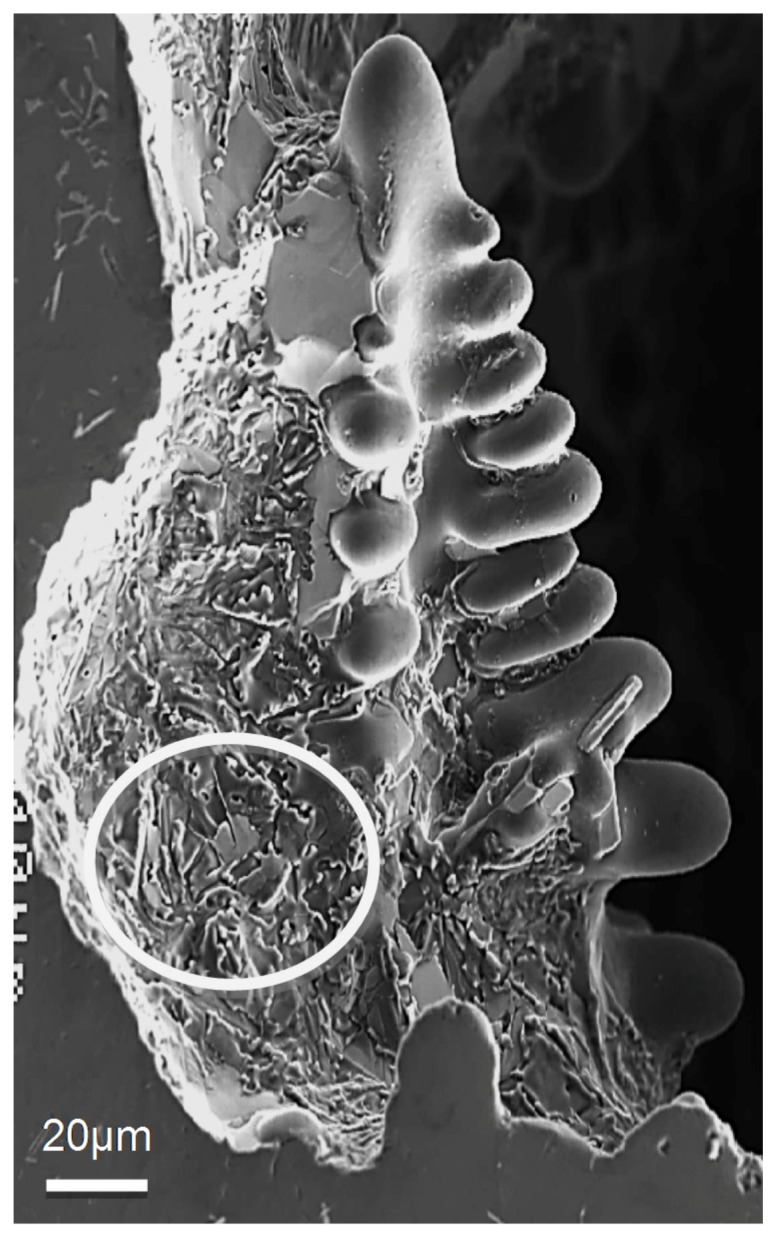
General view for the morphology of the eutectic Si particles in A1B alloy, DAS ~ 25 μm. The circled area shows thin flaky Si particles with rounded corners.

The values listed above reflect the modification effect of Sr and the partial modification effects of Mg and Be, but with increasing Fe levels it appears that most of the Be reacts with the Fe to possibly form a Be-Fe phase (Al_8_Fe_2_BeSi), thereby reducing the partial modification effect attributed to Be. These results are supported by the optical micrographs shown in Figure 1 and Figure 2. Figure 3 shows the morphology of the eutectic Si particles in the A1B alloy viewed within a pore. It is evident from the circled area that the particles are no longer acicular but rather in the form of thin flakes with rounded corners.

#### 3.1.2. High Solidification Rate (DAS ~ 25 μm)

**Figure 4:**
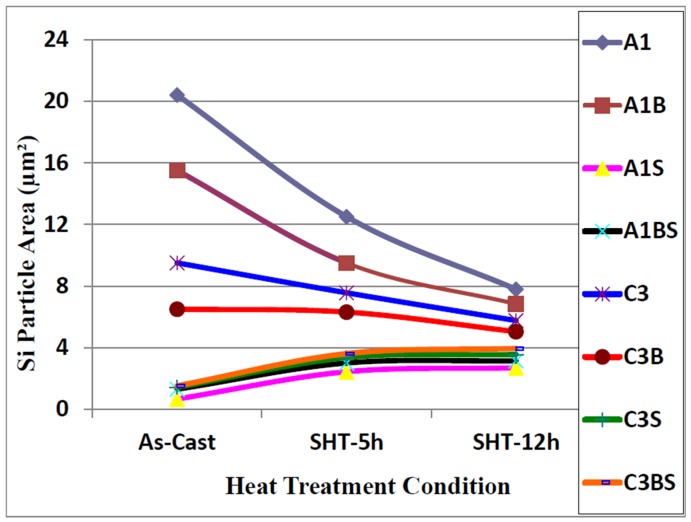
Variation in particle area of eutectic silicon as a function of heat treatment condition, DAS ~ 25 μm.

**Figure 5:**
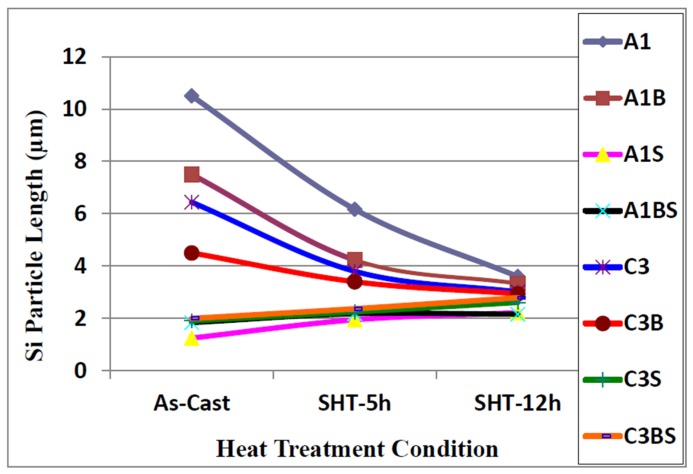
Variation in particle length of eutectic silicon as a function of heat treatment condition, DAS ~ 25 μm.

**Figure 6:**
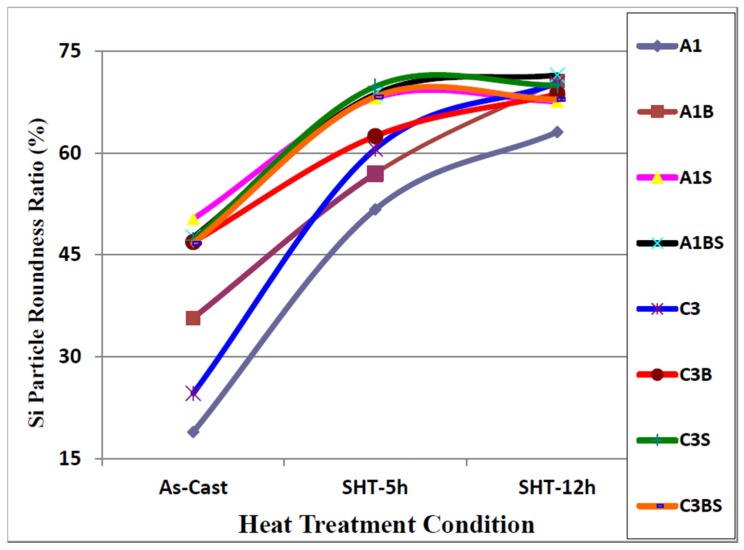
Variation in particle roundness of eutectic silicon as a function of heat treatment condition, DAS ~ 25 μm.

**Figure 7:**
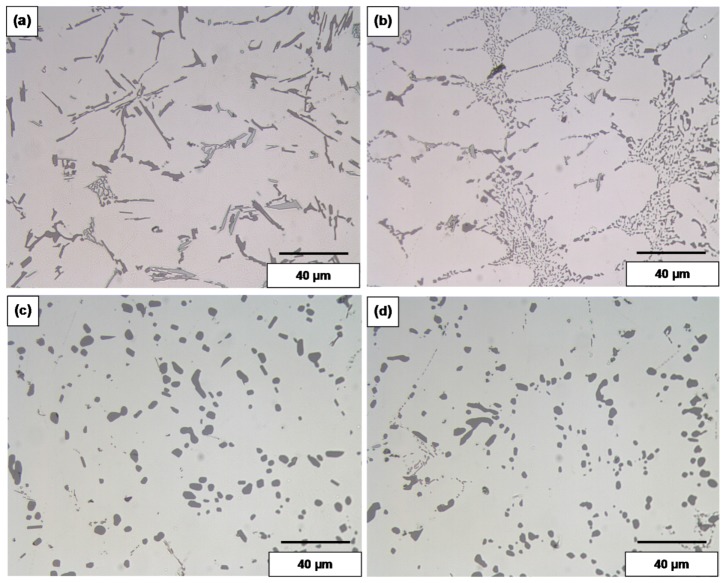
Morphology of eutectic silicon particles observed in the 357 alloys under the following conditions: (**a**) and (**c**) are the base alloy A1 in the as-cast and solution heat-treated conditions, respectively; (**b**) and (**d**) are A1BS alloy in the as-cast and solution heat-treated conditions, respectively, DAS of 25 μm.

Samples for microstructural analysis were sectioned from the tensile-tested bars ~10 mm below the fracture surface (away from the deformed portion of the tensile bar) to study each alloy in the as-cast and solution heat-treated conditions (540 °C/5 h and 540 °C/12 h). The dendrite arm spacing of these samples was about 24 μm. The Si particle characteristics of the as-cast alloys are presented in Table 3. Figure 4, Figure 5, Figure 6 and Figure 7 show the eutectic Si particle characteristics in the as-cast and solution heat-treated conditions for the eight alloys investigated.

**Figure 8:**
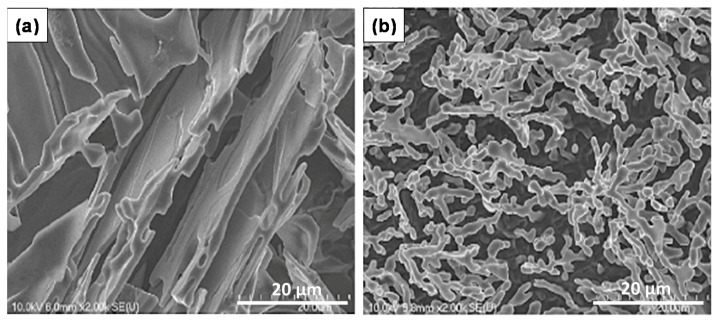
Scanning electron-micrographs of binary Al-7%Si: (**a**) graphite mold, non-modified; (**b**) graphite mold, modified with 54 ppm Sr [20].

Nafisi *et al*. [20] investigated eutectic nucleation in hypoeutectic Al-Si alloys. The nucleation mechanism of eutectic grains in hypoeutectic Al-Si foundry alloys has been investigated by examining deep etched specimens in high-resolution field emission gun scanning electron microscope (FEG-SEM). Figure 8 displays the Si particle morphology in non-modified and Sr modified Al-7%Si alloys. It is evident from this figure that the particle size depends on the way how the sample was sectioned from the casting. The addition of Be is well known to reduce oxide bifilm content in the cast alloy because the strengthening of the oxide on the liquid metal causes it to “hang up” from the lip of the ladle and so not enter the casting. The reduced bifilm content in the metal may explain the observed partial modification.

The Si particle measurements for the A1 base alloy, containing low Mg and low Fe, are listed in Table 4. In comparison, the average Si particle area decreased from 28.4 to 0.67 μm^2^, the particle length decreased from 15.5 to 1.25 μm, the aspect ratio decreased from 3.78 to 1.84, while the roundness increased from 18.9 to 50.3% in the Sr-modified alloy (alloy A1S). In the case of Be addition (alloy A1B), the particle area decreased to 10.3 μm^2^, the particle length to 6.6 μm, the aspect ratio to 2.73, while the roundness increased to 35.7%. A combined Sr and Be addition (alloy A1BS) resulted in decreasing the Si particle area to 1.31 μm^2^, the Si particle length to 1.84 μm, the aspect ratio to 1.94, and increased the roundness to 47.6%. Increasing the Mg and Fe content (alloy C3), resulted in decreasing the Si particle area to 6.9 μm^2^, the Si particle length to 6.44 μm, the aspect ratio to 3.01, while increasing the roundness to 24.6%. The combined effect of Sr, Be, Fe and Mg elements (alloy C3BS) resulted in decreasing the Si particle area to 1.53 μm^2^, the Si particle length to 2 μm, the aspect ratio to 1.99, and increasing the roundness to 46.7%. These values reflect the modification effect of Sr and the partial modification effects of Mg and Be.

**Table 4:** Eutectic silicon particle measurements for as-cast 357 alloy samples (DAS ~ 25 μm).

Alloy (Condition)	Area (μm^2^)		Length (μm)		Roundness (%)		Aspect Ratio	
	Av.	SD	Av.	SD	Av.	SD	Av.	SD
A1 (non-modified)	28.40	9.50	11.50	6.46	18.9	8.9	3.78	1.79
C3 (non-modified)	15.30	7.90	9.60	6.50	35.7	21.1	2.73	1.66
A1B (Be-modified)	6.90	3.40	6.44	4.35	44.6	10.5	2.01	1.07
C3B (Be-modified)	3.17	2.21	2.98	1.40	56.9	12.4	1.99	0.93
A1S (Sr-modified)	1.67	0.40	1.25	1.07	50.3	7.9	1.84	0.70
C3S (Sr-modified)	2.30	1.26	1.91	0.88	59.2	9.7	1.99	0.92
A1BS (Be + Sr-modified)	1.61	1.04	1.84	1.66	47.6	8.8	1.94	0.81
C3BS (Be + Sr-modified)	1.53	0.40	2.00	1.76	46.7	8.9	1.99	0.93

Codes A and C correspond to the Fe levels 0.09 and 0.6, respectively; while codes 1 and 3 correspond to the Mg levels 0.4 and 0.8, respectively; as well as both codes B and S represent Be additions and Sr modification, respectively.

For high Fe- and Mg-containing alloys, the simultaneous addition of Be and Sr has less of an effect on the Si particle characteristics than addition of each element individually. The combined effect of adding the four elements can be confirmed from an analysis of the microstructures and data given in Table 3 and Table 4. Comparing the data in Table 3 and Table 4 shows that increasing the solidification rate causes a significant improvement in the Si particle characteristics for alloys containing Be, Sr or Be + Sr, as expected. The observed increase in the roundness and aspect ratio of Si particles reported in Table 3 for non-modified A1 and C3 alloys may be attributed to the longer solidification time before the samples reach room temperature.

After applying solution heat treatment, as shown in Figure 4 through Figure 7, the results observed may be interpreted as follows: the fragmentation and spheroidization of the eutectic Si particles in the 357 base alloy A1 occurs as the alloy is subjected to solution heat treatment and continues as the solution treatment time increases from 5 to 12 h, as reflected in the continual decrease in the Si particle size and a corresponding increase in the particle roundness. The influence of alloying additions on the degree of refinement of the Si phase may be discerned from the particle characteristics observed for the various alloys in the as-cast condition. The greatest refinement is obtained for the Sr-modified alloy A1S with A1BS and C3BS alloys displaying a close degree of refinement, whereas the addition of Be alone reduces the particle size in both A1B and C3B alloys, but not to the extent as that observed with a combined Be + Sr addition. Applying solution heat treatment thereafter produces only a very slight decrease in particle size, however, a slight increase is obtained for alloys A1BS and C3BS; this increase may be attributed to the coarsening of the Si particles with the increase in solution treatment time [16,17,18,20].

**Figure 9:**
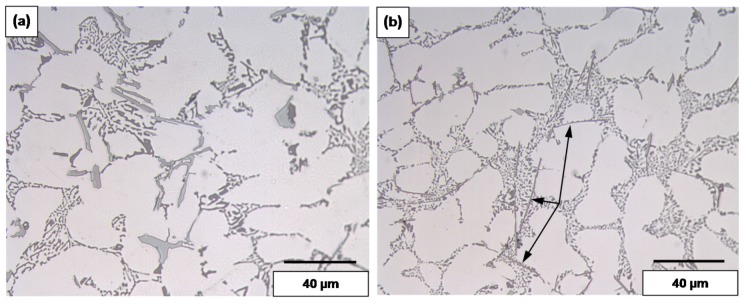

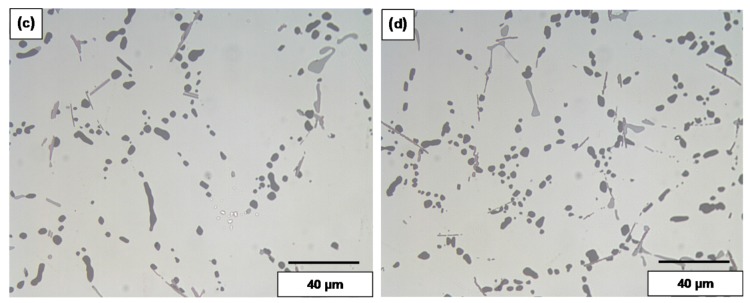
Morphology of eutectic silicon particles observed in the 357 alloys under the following conditions: (**a**) and (**c**) are the high levels of Mg and Fe alloy C3 in the as-cast and solution heat-treated conditions, respectively; (**b**) and (**d**) are Sr-modified C3BS alloy with Be in the as-cast and solution heat-treated conditions, respectively, DAS of 25 μm; black arrows in (**b**) point to precipitation of β-Al_5_FeSi platelets.

**Figure 10:**
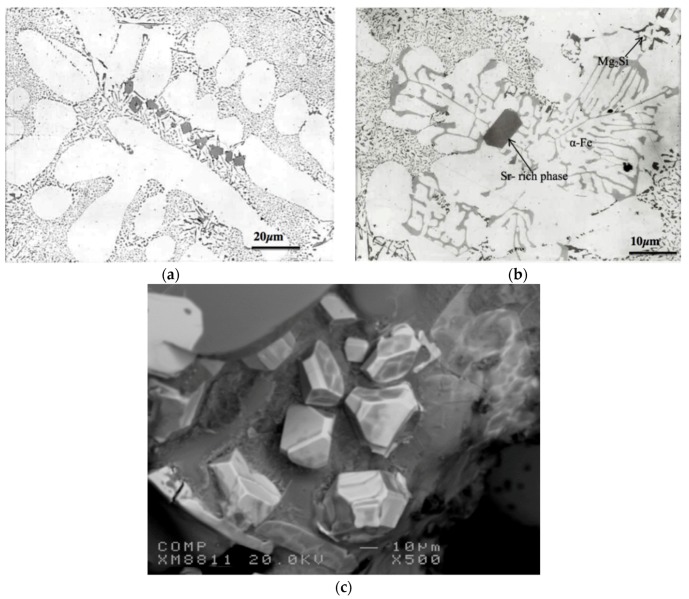
Morphlogy of eutectic Si particles in A1S alloy in the as cast condition: (**a**) segregation of the Sr-rich particles; (**b**) formation of α-Fe and Sr-rich phases; (**c**) an example of the tetrahedral shape of the Al_4_SrSi_2_ particles, DAS ~ 25 μm.

Figure 9 and Figure 10 depict the microstructures of selected as-cast and solution heat treated samples of these alloys, to illustrate the changes in the morphology and size of the Si particles, depending on the alloying additions and heat treatment conditions. Increasing the solutionizing time from 5 to 12 h also leads to further improvement in the morphology of eutectic Si particles. The particle area in the C3 alloys containing higher levels of Mg and Fe was seen to decrease while in the A1 alloys with low Mg and low Fe, the particle area was seen to increase because of the coarsening brought about in these particles following solution treatment for 12 h at 540 °C. Spheroidization of the Si particles may be clearly noted in Figure 8c,d and Figure 9c,d, for the long solution treatment time [21,22]. 

**Figure 11:**
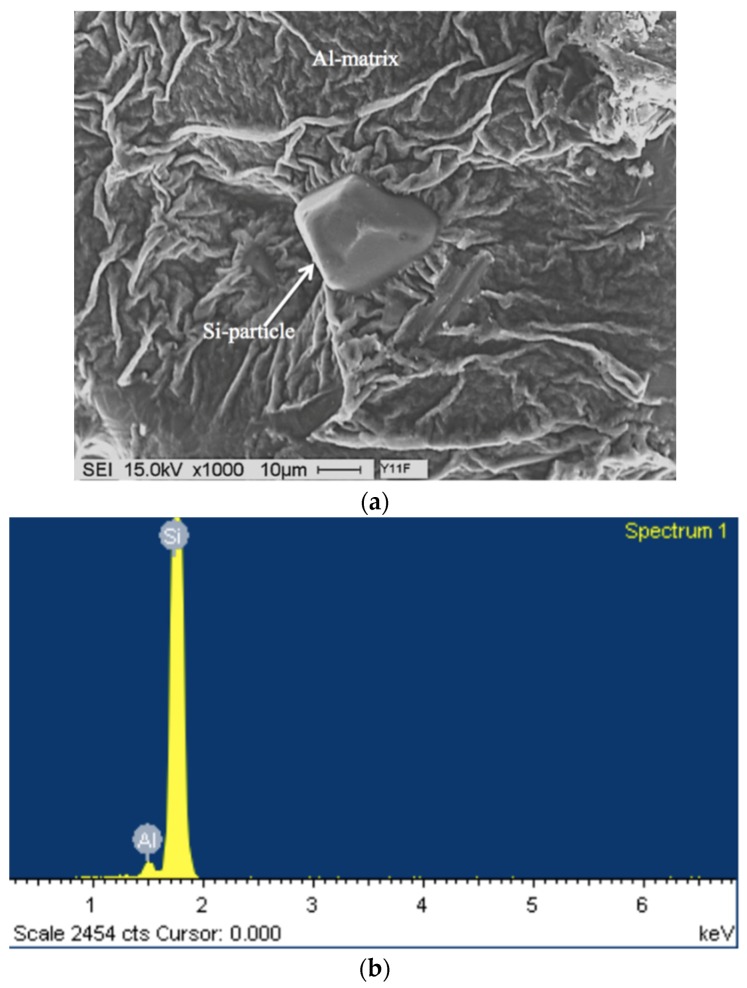
(**a**) Secondary electron image of the fracture surface of A1B alloy solutionized at 560 °C for 12 h, DAS ~ 25 μm; (**b**) EDS spectrum.

Compared to the microstructure of the as-cast base alloy A1 shown in Figure 11a, the as-cast A1BS alloy sample displays on the whole fine Si particles in the interdendritic regions. The large standard deviation observed in Table 3 is due the presence of large Si particles (see solid arrow in Figure 8 mixed with the fine Si particles.

### 3.2. Overmodification and Overheating

When Sr is added in proper amounts, eutectic Si particles precipitate in ultrafine form as displayed in Figure 10a. Exceeding that concentration would lead to precipitation of Sr in the form of primary particles without causing noticeable microstructural defects or coarsening of the eutectic Si particles as shown in Figure 10b [23]. It is interesting to note that the Sr-rich particles are rejected to the outer edges of the eutectic Si colonies. According to Mohamed and Samuel [24], an evidence of Sr over-modification is the appearance of Sr-containing intermetallic phases in the microstructure such as the Al_4_SrSi_2_ particles. The presence of the Sr-rich particles would reduce the properties of the alloy causing them revert to values similar to those obtained from non-modified alloys.

Figure 10c exhibits an example of the tetrahedral shape of the Al_4_SrSi_2_ particles. It was observed that in the case of over-modification, precipitation of Al_4_SrSi_2_ phase particles is not only confined to eutectic Si areas, they also appear on the pre-existing Fe-based intermetallic particles (Figure 10b).

Increasing the solutionizing temperature to 560 °C, a temperature approximately 5 °C below the eutectic formation temperature, would lead to incipient melting of the matrix as shown in Figure 11. In this case the eutectic Si particles appear in the form of tetrahedral rather than spherical as viewed in Figure 9 (presumably formed on an oxide film). The associated EDS spectrum is presented in Figure 11b, revealing a strong peak due to Si which is expected from relatively large Si particles (~10 μm). The presence of such a structure would cause great deterioration in the alloy mechanical properties (maximum attainable strength is about 50 MPa with elongation less than 0.5%) [10].

## 4. Fractography

In order to arrive at a better understanding of the importance of the Si morphology, tensile bars prepared from Be-treated alloys coded A1B and A1BS were tested in the as-cast and solution heat-treated (at 540 °C) conditions. Table 5 lists the tensile properties of A1, A1B and A1BS alloys. It is evident from the data reported in this table that addition of Be has a good effect on the alloy ductility. The drop in ductility in the A1S alloy may be attributed to the presence of porosity [25,26,27]. Simultaneous addition of Be and Sr seems to minimize the amount of porosity in the A1BS alloy, hence, the observed improvement in the percent elongation.

**Table 5:** Tensile properties of A1, A1B, A1S, A1BS alloys in the as-cast and solution heat treated condition.

Alloy Code	Mechanical Property	As-cast	SHT 5 h	SHT 12 h
A1	UTS (MPa)	203.8 ± 8.3	262.2 ± 8.5	255.0 ± 8.6
	YS (MPa)	97.1 ± 3.5	115.9 ± 5.8	107.5 ± 5.8
	El (%)	5.5 ± 0.65	14.9 ±1.2	15.6 ± 1.4
A1B	UTS (MPa)	195.9 ± 9.2	252.0 ± 8.4	270.5 ± 9.2
	YS (MPa)	98.0 ± 5.6	111.5 ± 6.8	120.5 ± 7.5
	El (%)	7.0 ± 0.82	17.8 ±1.4	21.6 ± 1.4
A1S	UTS (MPa)	205.3 ± 9.3	251.9 ± 8.5	257.4 ± 8.5
	YS (MPa)	96.7 ± 5.8	114.4 ± 4.5	116.3 ± 6.4
	El (%)	7.6 ± 0.67	16.8 ± 1.3	18.1 ± 1.5
A1BS	UTS (MPa)	198.4 ± 7.5	265.7 ± 8.3	272.5 ± 9.1
	YS (MPa)	106.9 ± 4.5	124.2 ± 6.5	135.2 ± 6.5
	El (%)	5.6 ± 0.36	18.1 ± 1.3	23.7 ± 1.5

SHT = Solution heat treatment; UTS = ultimate tensile strength; YS = yield strength; and El = elongation.

**Figure 12:**
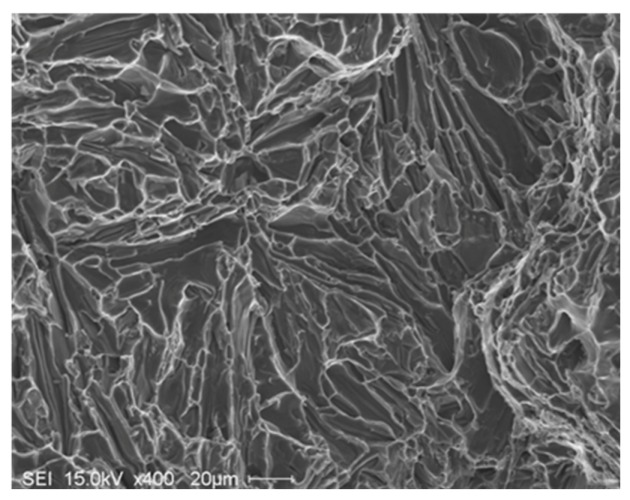
Fracture surface of the A1 alloy in the as cast condition, DAS ~ 25 μm.

**Figure 13:**
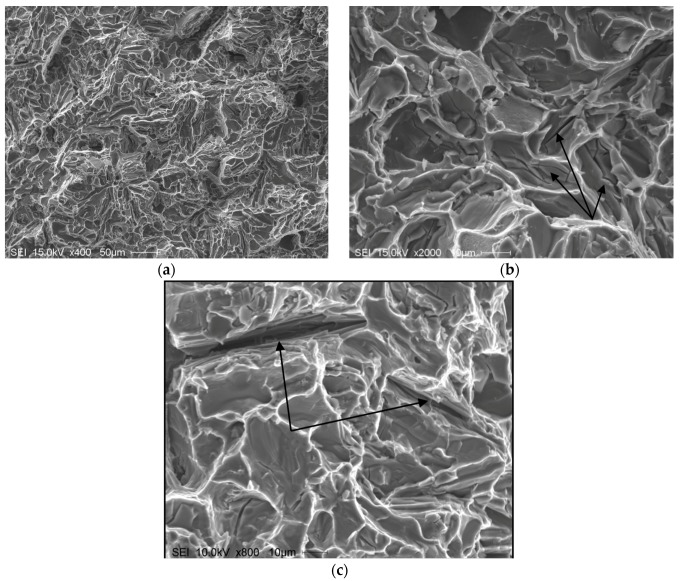
Fracture surface of A1B alloy in the as cast condition: (**a**) general view; (**b**) high magnification image, DAS ~ 25 μm; black arrow in (**b**) show fracture planes through the bifilm cracks in the interiors of Si particles; (**c**) typical bifilm oxides (arrowed).

**Figure 14:**
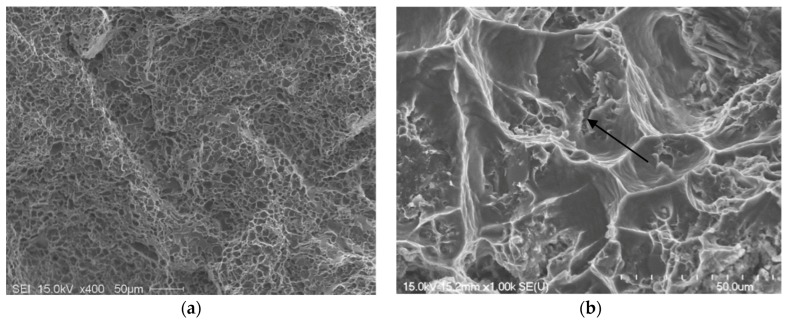
Fracture surface of A1BS alloy: (**a**) as cast contion; (**b**) after solutionizing at 540 °C for 12 h, DAS ~ 25 μm; the arrow in (**b**) refers to central cracks from failed Si particles

Figure 12 presents the shape of the fractured Si particles in the non-modified A1 alloy. As can be seen, the Si particles appear to have a fan-like morphology. Although Figure 11a shows the fracture surface of A1B in the as-cast condition as being composed of long Si particles branched in differenced directions (see white circled area), the size of the Si particles are relatively much smaller than those observed in Figure 12. The high magnification electron micrograph shown in Figure 13 reveals the cracking of the non-modified Si particles during tensile testing. Figure 14a exhibits a significant change in the fracture details of Sr modified A1B alloy (*i.e.*, A1BS alloy) where a uniform dimple structure has replaced the long particles previously observed in Figure 12 and Figure 13. The central cracks (black arrow-Figure 14b) from failed Si particles are surrounded by much larger areas of matrix which have plastically sheared to cusp-like morphology, forming the “ductile” dimples.

**Figure 15:**
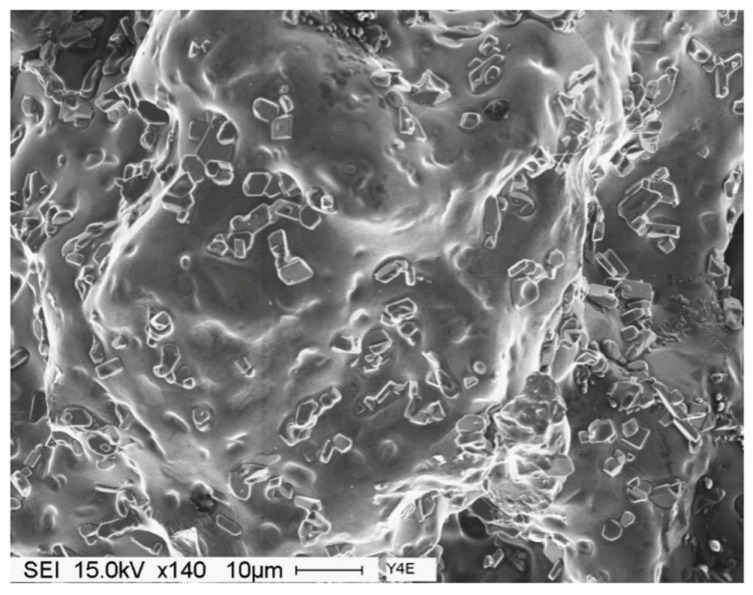
Secondary electron image showing the fracture surface of A1B alloy solutionized at 560 °C for 12 h, DAS ~ 25 μm.

Solutionizing the A1BS alloy for 12 h at 540 °C results in the formation of deep dimples with clear deformations lines on their surfaces, Figure 14b, indicating a marked improvement in the alloy ductility (Table 4). The fracture surface of the overheated A1B alloy is shown in Figure 15, where the eutectic Si particles are tetrahedral shaped. This type of fracture which has locally remelted while adjacent to an open void, allowing the melted liquid to form its smooth contour.

**Figure 16:**
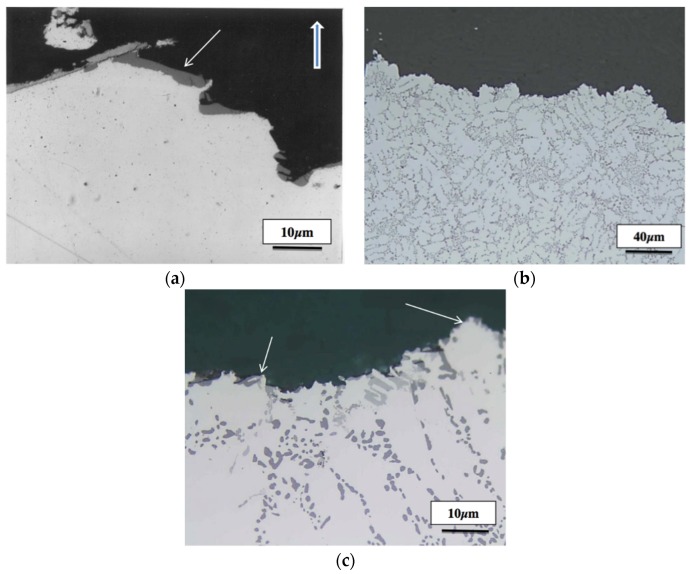
Microstructure beneath the fracture surface of: (**a**) A1alloy-as cast; (**b**) A1BS alloy-as cast; (**c**) A1BS alloy after solutionizing at 540 °C for 12 h. The thick white arrow in (**a**) indicates the tensile direction, whereas thin arrows in (**a**) and (**c**) show the tips of the dendrites, DAS ~ 25 μm.

The microstructures beneath the fracture surfaces of A1 and A1BS are presented in Figure 16. The presence of long Si particles in A1 alloy following casting seems to accelerate the crack propagation as shown in Figure 16a. Addition of Sr to A1B alloy resulted in elongated dendrites aligned in the tensile direction with practically no visible secondary cracks beneath the primary one, Figure 16b. Solutionizing the A1BS alloy at 540 °C alloy for 12 h (Figure 14c), further emphasizes this observation (white arrows).

## 5. Conclusions

Based on the results presented in this study, the following conclusions may be drawn:

1. In non-modified alloys, the average values of the Si parameters are more or less equal to standard deviations. Chemical modification (specially with addition of 150 ppm Sr) found to reduce this difference to a great extent.
2. Beryllium causes partial modification of the eutectic Si particles, similar to that reported for Mg addition.
3. The addition of 0.8% Mg reduced the eutectic temperature by ~10 °C, *i.e.*, 1.3 °C per 0.1% of Mg addition. Interactions between Fe-Be and Sr-Mg may reduce the modification efficiency of Be and Sr.
4. The presence of long, acicular Si particles in the matrix accelerates crack propagation, leading to poor ductility. Additionally, the possibility of the existence the planar bifilm cracks in the Si particles may accelerate fracture.
5. Depending on the solidification rate, full modification of the eutectic Si may be achieved by the addition of 150–200 ppm of Sr. Exceeding this concentration leads to the precipitation of Sr in the form of primary Al_4_SrSi_2_ particles which have a negative influence on the alloy tensile properties. The Sr amount may be reduced through reducing the oxide films.
6. Exceeding the proper solution temperature would lead to incipient melting of the aluminum matrix with a change in the eutectic Si particles from spherical to tetrahedral form.
7. In low iron (less than 0.1 wt%) Al-Si-Mg alloys, the mechanical properties in the as-cast and heat-treated conditions are mainly controlled by the eutectic Si particle charactersitics. Increasing the iron content, and hence the volume fraction of Fe-based intermetallics, leads to a complex failure mode.

## References

[1] Liao C., Chen J., Pan C. (2012). Microstructure of Al4Sr phase in Al-Sr master alloy and its effect on modification properties. Procedia Eng..

[2] Davis J.R. (1994). ASM Specialty Handbook: Aluminum and Aluminum Alloys.

[3] Sokolowski J.H., Djurdjevic M.B., Kierkus C.A., Northwood D.O. (2001). Improvement of 319 aluminum alloy casting durability by high temperature solution treatment. J. Mater. Process. Technol..

[4] Sarada B.N., Srinivasamurthy P.L. (2013). Swetha, microstructural characteristics of Sr And Na modified Al-Mg-Si alloy. Int. J. Innov. Res. Sci. Eng. Technol..

[5] Shivkumar S., Keller C., Trazzera M., Apelian D. Precipitation Hardening in A356 Alloys. Proceedings of the International Symposium on Production, Refining, Fabrication and Recycling of Light Metals.

[6] Cáceres C.H., Davidson C.J., Griffiths J.R., Wang Q.G. (1999). The effect of Mg on the microstructure and mechanical behavior of Al-Si-Mg casting alloys. Mater. Metall. Trans. A.

[7] Hatch J.E. (1984). Aluminum: Properties and Physical Metallurgy.

[8] Murali S., Raman K.S., Murthy K.S.S. (1995). The formation of β-phase and Be-Fe phases in Al-7Si-0.3Mg alloy containing Be. Mater. Sci. Eng. A.

[9] Kammer C. (1999). Aluminum Handbook, Vol. 1: Fundamentalsand Materials.

[10] Han Y., Samuel A.M., Doty H.W., Valtierra S., Samuel F.H. (2014). Optimizing the tensile properties of Al-Si-Cu-Mg 319-type alloys: Role of solution heat treatment. Mater. Des..

[11] Samuel E., Samuel A.M., Doty H.W., Valtierra S., Samuel F.H. (2014). Intermetallic phases in Al-Si based cast alloys: New perspective. Int. J. Cast Metals Res..

[12] Apelian D., Shivkumar S., Sigworth G. (1989). Fundamental aspects of heat treatment of cast Al-Si-Mg alloys. AFS Trans..

[13] Roy E.L. (1992). Castings.

[14] Samuel F.H., Liu H., Samuel A.M. (1993). Effect of melt cleanliness on the properties of an Al-10 wt% Si-10 Vol% SIC(P) composite. Metall. Trans. A Phys. Metall. Mater. Sci..

[15] Tiryakioğlu M., Campbell J., Nyahumwa C. (2011). Fracture surface facets and fatigue life potential of castings. Metall. Mater. Trans. B.

[16] Campbell J. (2006). An overview of the effects of bifilms on the structure and properties of cast alloys. Metall. Mater. Trans. B.

[17] Cho Y.H., Lee H.-C., Oh K.H., Dahl A.K. (2008). Effect of strontium and phosphorus on eutectic Al-Si nucleation and formation of β-Al5FeSi in hypoeutectic Al-Si foundry alloys. Metall. Mater. Trans. A.

[18] Ibrahim M.F. (2015). Microstructural characterization of beryllium treated Al-Si alloys. Adv. Mater. Sci. Eng..

[19] (2015). Standard Specification for Aluminum-Alloy Permanent Mold Castings.

[20] Nafisi S., Ghomashchi R., Vali H. (2008). Eutectic nucleation in hypoeutectic Al-Si alloys. Mater. Charact..

[21] Ma Z., Samuel A.M., Doty H.W., Valtierra S., Samuel F.H. (2014). Effect of Fe content on the fracture behaviour of Al-Si-Cu cast alloys. Mater. Des..

[22] Taylor J.A., StJohn D.H., Zheng L.H., Edwards G.A., Barresi J., Couper M.J. (2001). Solution treatment effects in Al-Si-Mg casting alloys: Part 1—Intermetallic phases. Alum. Trans..

[23] Dasgupta R., Brown C.G., Mark S. (1988). Analysis of overmodified 356 aluminum alloy. AFS Trans..

[24] Mohamed A.M.A., Samuel F.H. (2012). A review on the heat treatment of Al-Si-Cu/Mg casting alloys. Heat Treatment: Conventional and Novel Applications.

[25] Elsharkawi E.A., Samuel E., Samuel A.M., Samuel F.H. (2010). Effects of Mg, Fe, Be additions and solution heat treatment on the pi-AlMgFeSi iron intermetallic phase in Al-7Si-Mg alloys. J. Mater. Sci..

[26] Ibrahim M.F. (2015). Effects of Be, Sr, Fe and Mg Interactions on the Microstructure and Mechanical Properties of Aluminum Based Aeronautical Alloys. Ph.D. Thesis.

[27] Wang Q.G. (2003). Microstructural effects on the tensile and fracture behavior of aluminum casting alloys A356/357. Metall. Mater. Trans. A.

